# In search of suitable reference genes for gene expression studies of human renal cell carcinoma by real-time PCR

**DOI:** 10.1186/1471-2199-8-47

**Published:** 2007-06-08

**Authors:** Monika Jung, Azizbek Ramankulov, Jan Roigas, Manfred Johannsen, Martin Ringsdorf, Glen Kristiansen, Klaus Jung

**Affiliations:** 1 Department of Urology, Charité – Universitätsmedizin Berlin, Campus Charité Mitte, Charitéplatz 1, 10117 Berlin, Germany; 2 Institute of Pathology, Charité – Universitätsmedizin Berlin, Campus Charité Mitte, Charitéplatz 1, 10117 Berlin, Germany; 3 Republic Center of Urology, Bishkek, Kyrgyz Republic

## Abstract

**Background:**

Housekeeping genes are commonly used as endogenous reference genes for the relative quantification of target genes in gene expression studies. No conclusive systematic study comparing the suitability of different candidate reference genes in clear cell renal cell carcinoma has been published to date. To remedy this situation, 10 housekeeping genes for normalizing purposes of RT-PCR measurements already recommended in various studies were examined with regard to their usefulness as reference genes.

**Results:**

The expression of the potential reference genes was examined in matched malignant and non-malignant tissue specimens from 25 patients with clear cell renal cell carcinoma. Quality assessment of isolated RNA performed with a 2100 Agilent Bioanalyzer showed a mean RNA integrity number of 8.7 for all samples. The between-run variations related to the crossing points of PCR reactions of a control material ranged from 0.17% to 0.38%. The expression of all genes did not depend on age, sex, and tumour stage. Except the genes TATA box binding protein (*TBP*) and peptidylprolyl isomerase A (*PPIA*), all genes showed significant differences in expression between malignant and non-malignant pairs. The expression stability of the candidate reference genes was additionally controlled using the software programs geNorm and NormFinder. *TBP *and *PPIA *were validated as suitable reference genes by normalizing the target gene *ADAM9 *using these two most stably expressed genes in comparison with up- and down-regulated housekeeping genes of the panel.

**Conclusion:**

Our study demonstrated the suitability of the two housekeeping genes *PPIA *and *TBP *as endogenous reference genes when comparing malignant tissue samples with adjacent normal tissue samples from clear cell renal cell carcinoma. Both genes are recommended as reference genes for relative gene quantification in gene profiling studies either as single gene or preferably in combination.

## Background

Gene expression studies in tumour tissue in comparison with its corresponding normal tissue counterpart open up prospects for identifying new biomarkers and targets characteristic of the respective tumour entity. For that purpose, the relative quantification of reverse transcription-PCR (RT-PCR) data is the method of choice to ascertain gene expression results [1,2]. This method is based on the normalization of the target gene expression on any stably expressed internal reference gene, a so-called "housekeeping" gene, measured in the same biological material. A crucial problem involved here is finding such a suitable reference gene which has to be tested and verified under defined study conditions. The general conditions a reference gene must meet is that its expression in the samples studied be stable, non-regulated and constant and not be influenced by biological (e.g., age, gender, metabolism, disease stage) or experimental (e.g., addition or deprivation of physiological, physical or chemical agents) conditions or treatments [3-6]. In addition, the reference gene and the target gene should have similar ranges of expression to avoid analytical problems.

In literature, several single housekeeping genes or housekeeping gene indexes summarizing two or more housekeeping genes have been used for relative quantification [4,5,7,8]. However, these genes were often adopted without exact knowledge of their individual expression behaviour under the special study conditions. Recently, we discussed that issue in two studies concerning the identification of candidate reference genes for the relative quantification of expression data in the urological tumours of the prostate and bladder [8,9].

Renal cell carcinoma (RCC) is another important urological tumour. In 2007, RCC is estimated to cause 51.190 new cases and 12.890 deaths in the USA [10]. RCC is, in most cases, clinically asymptomatic and casually detected by routine ultrasonographic follow-ups in persons otherwise in generally good health [11]. The clear cell subtype of RCC (ccRCC) is the most frequent malignant RCC showing an incidence of about 75% [12]. It has a worse prognosis in comparison with the papillary and chromophobe RCC subtypes that account for 10% and 5% of RCC, respectively [12]. Although numerous tumour markers in RCC were tested in the past, to date no definitive biomarkers are available for diagnosis, monitoring, and predicting the outcome of RCC [13]. Thus, there is an urgent need to search for new markers. To identify potential new candidates gene expression profiles using the DNA microarray technique and subsequent RT-PCR assays are helpful tools [14]. Therefore, we searched for suitable normalization genes for gene expression studies on RCC tissue samples. Our strategy was the same as that used in studies of prostate and bladder normalization gene findings [8,9]. Using the MeSH terms "renal cell carcinoma", "gene expression", and "RT-PCR" combined with the Boolean operator "AND" we performed a PubMed search of articles published from October 1993 to August 2006. We had access to 156 articles that used 19 various reference genes (Table 1). It was remarkable that beta-actin (*ACTB*; 64 times; 37%) and glyceraldehyde-3-phosphate dehydrogenase (*GAPDH*; 63 times; 36%) were used as normalizer genes in about three quarters of all studies. The use of these two genes for normalization is historically grown. Although their regulated expression shown under different conditions is inconsistent with their use as normalizers [1,15], this fact has often been disregarded till now or at least it has not been checked under the study conditions [15-18]. All other housekeeping genes cited accounted for only 1 to 6%. Only a few studies compared or used more than one reference gene, often in other organisms [19-21] or under other clinical conditions [7,22,23]. This literature search clearly proves that an univocal reference gene for gene expression studies in renal cell carcinoma does not exist. The search results additionally emphasize the need for a systematic study to identify suitable reference genes for gene expression studies in renal cell carcinoma.

**Table 1:** Reference genes used for gene expression studies in renal cell carcinoma

**Gene name**	**Publications using that gene as reference gene**	
	
	**Number**	**%**
***GAPDH***	63	36.4
***ACTB***	64	37.0
18S ribosomal RNA	10	5.8
Beta-2-microglobulin	7	4.0
***RPLPO***	5	2.9
***HMBS***	3	1.7
***HPRT1***	3	1.7
***PPIA***	3	1.7
***TBP***	3	1.7
Alpha tubulin	3	1.7
***ALAS1***	1	0.6
Enolase 1, (alpha)	1	0.6
Beta globin	1	0.6
Karyopherin alpha 6	1	0.6
Membrane cofactor protein	1	0.6
Phosphoglycerate kinase 1	1	0.6
Ribosomal protein L7	1	0.6
Ribosomal protein S9	1	0.6
Ribosomal protein S14	1	0.6

Results of a PubMed search from October 1993 to August 2006 with 156 articles and 173 descriptions of different reference genes. Candidate reference genes tested in the present study are given as gene symbols in bold letters and their corresponding full names and accession numbers are indicated in Table 2. Further comments concerning the search results see the section "Limitations of this study".

The aim of this study was to identify suitable reference genes for the purpose of normalization in RCC gene expression studies. Therefore, we examined a panel of 10 candidate reference genes listed in Table 2 with regard to their expression behaviour in 25 matched malignant and non-malignant RCC tissue samples. This investigation was focused on the clear cell RCC subtype as the most frequent malignant RCC. We selected as potential reference genes either genes taken from the above-mentioned literature search or genes that were shown to be appropriate for normalization in previous studies of other tissues [6,8,9]. The suitability as reference gene was estimated by comparing the expression stability of the respective gene in malignant and non-malignant tissue using different mathematical procedures and computer programs. The use of unsuitable reference genes in the normalization procedure could result in serious errors in gene expression studies, which could lead to wrong conclusions being drawn [8,24-26]. These aspects are illustrated by the example of the relative gene quantification of "a disintegrin and metalloproteinase domain 9" (*ADAM9*) using up- and down-regulated housekeeping genes as normalizers.

**Table 2:** Characteristics of gene-specific real-time PCR assays

Gene symbol	Gene name	Accession No.	Primer/Probe Sequence [5'→3']	Amplicon Size [bp]	Detection Dye/Probe	PCR Efficiency^d^
*ACTB*	Actin, beta	NM_001101	Forward: agcctcgcctttgccga Reverse: ctggtgcctggggcg Probe: **F**-ccgccgcccgtccacacccgccT**-P**	174	TM^a^	1.89
*ALAS1*	5-Aminolevulinate delta-, synthase 1	NM_000688	LightCycler-h-ALAS Housekeeping Gene Set Roche (Cat. No. 03 302 504 001)	127	Hyb^b^	1.97
G*APDH*	Glyceraldehyde-3-phosphate dehydrogenase	NM_002046	Forward: gaaggtgaaggtcggagtc Reverse: gaagatggtgatgggatttc Probe: **F**-caagcttcccgttctcagccT**-P**	226	TM	1.99
*HMBS*	Hydroxymethyl-bilane synthase *Alias: *Porphobilinogen deaminase (PBGD)	NM_000190	LightCycler-h-PBGD Housekeeping Gene Set Roche, Cat. No. 03 146 073 001	150	Hyb	1.98
*HPRT1*	Hypoxanthine phosphoribosyl transferase 1	NM_000194	LightCycler-h-HPRT Housekeeping Gene Set Roche (Cat. No. 03 261 891 001)	181	Hyb	2.00
*PPIA*	Peptidylprolyl isomerase A *Alias: *Cyclophilin A	NM_021130	Hs_PPIA_1_SG QuantiTect Primer Assay Qiagen (Cat. No. QT00052311)	121	SGI^c^	1.84
*RPLPO*	Ribosomal protein, large, PO	NM_001002	Hs_RPLPO_1_SG QuantiTect Primer Assay Qiagen (Cat. No. QT00075012)	79	SGI	1.92
*SDHA*	Succinate dehydrogenase complex, subunit A, flavoprotein (Fp)	NM_004168	Forward: cactggaggaagcacaccc Reverse: ccttcccagtgccaacgtccacaat Probe: **F**-ccttcccagtgccaacgtccacaaT**-P**	78	TM	1.92
*TBP*	TATA box binding protein	NM_003194	Forward: ttcggagagttctgggattgta Reverse: tggactgttcttcactcttggc Probe: **F**-ccgtggttcgtggctctcttatcctcaT**-P**	227	TM	1.88
*TUBB*	Tubulin, beta	NM_178014	Hs_TUBB_1_SG QuantiTect Primer Assay Qiagen (Cat. No. QT00089775)	120	SGI	1.98
*ADAM9*	ADAM metallopeptidase domain 9, (meltrin gamma)	NM_003816	Forward: ggtgacagatttggcaattgtg Reverse: ttgtgccttcgttaaccatcc Donor probe: acgcctagtcgaggcaccaaatgttg-6Fl Acceptor probe: Cy5.5-gtgtggatttccagctaggatcagatgttcc-P	226	Hyb	1.95

^a^TM = TaqMan probes are 3'-end F, FAM (6-carboxy-fluorescein) and near 5'-end T, TAMRA (6-carboxy-tetramethyl-rhodamine) labelled and P, phosphorylated

^b^Hyb = Hybridization probes, two different labelled probes are included in the kit (Roche); for target gene see text

^c^SGI = SYBR^® ^Green I fluorescent dye

^d^PCR efficiencies were calculated according to Rasmussen [2].

## Results

### Assessment of preanalytical and analytical variables

Preanalytical variables like the collection and storage of samples and the process of RNA isolation determine the quality of RNA that is used for subsequent quantitative expression analyses. RNA quality, as a summary parameter, characterizes possible negative effects with regard to deficient procedures of collection and preparation of tissue samples. Therefore, the isolated RNA samples from the 25 matched malignant and non-malignant samples were characterized with regard to their concentration, purity, and integrity. Only RNA samples with high quality should be included in this study to avoid erroneous conclusions. However, all RNA samples isolated from renal tissue specimens preserved in RNAlater solution exhibited a high quality. The mean A_260/280 _ratio of RNA samples was 2.05 ± 0.027 (range from 1.99 to 2.12.) and reflected pure and protein-free RNA. The RNA integrity as an essential quality criterion was characterized by the so-called RNA integrity number (RIN) measured on the Agilent 2100 Bioanalyzer. The mean RIN value (± SD) of all RNA samples was 8.7 ± 0.77 (range from 7.0 to 10.0). The matched malignant and non-malignant tissue samples reached mean RIN values (and SD) of 9.09 ± 0.57 and 8.32 ± 0.75, respectively, whose difference was significant (paired Student's t test; P < 0.001). Preceding orienting comparisons of RIN values measured in RNA samples isolated from paired tissue specimens stored in RNAlater solution or snap-frozen in liquid nitrogen immediately after surgery showed significantly higher values obtained using the former manner of preservation (n = 10; mean RIN value and SD of 8.21 ± 1.02 vs. 5.42 ± 2.80; P < 0.004). We therefore exclusively used RNAlater solution for tissue preservation.

There was a strong correlation between the RIN values of the paired samples (r_s _= 0.635, P < 0.001). All gene expression levels both in the malignant and non-malignant samples did not correlate with the RIN number (r_s _= -0.012 to 0.325; P = 0.956 to 0.113) as it was shown as proof for intact, high quality RNA [27].

Pooled cDNA samples were used as precision control materials for each gene-specific PCR run. These control materials were adjusted to the ranges of Cp values that were characteristic for the particular genes in the tissue samples. Intra-run and between-run analytical performances of the RT-PCR measurements were determined using these control materials. The intra-run precision (n = 12) was 0.26% and 3.56% for *ACTB *amplifications with a mean Cp-value of 21.26 and the corresponding mean concentration of 4.59 arbitrary units, respectively. The between-run variations (n = 5) ranged from 0.17% (Cp mean 25.63 ± 0.04 of *SDHA *control cDNA) as the lowest to 0.38% (Cp mean 26.46 ± 0.10 of *HMBS *control cDNA) as the highest value. Corresponding coefficients of variation for the concentrations data ranged from 2.26% to 10.9%.

### Expression levels of candidate reference genes

**Figure 1:**
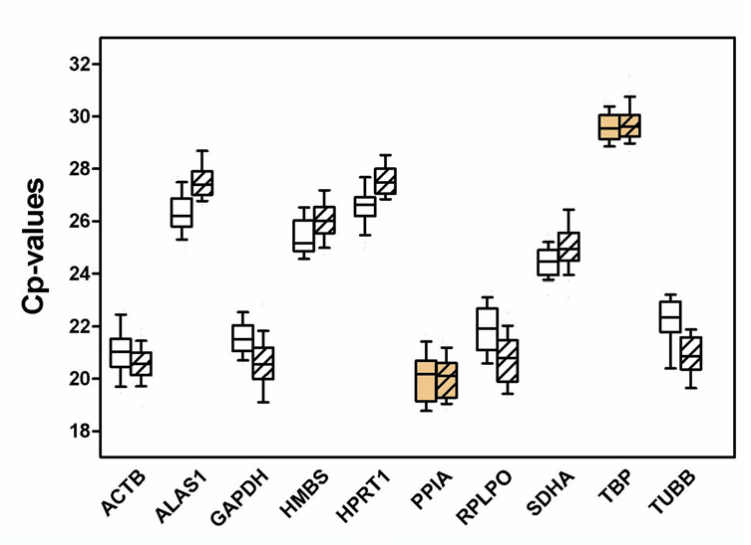
**Expression levels of candidate reference genes in non-malignant and malignant renal cell carcinoma samples**. Values are given as real-time PCR crossing points (Cp) cycle numbers. Boxes (blank: non-malignant; cross striated: malignant) represent the lower and upper quartiles with medians; whiskers illustrate the 10 to 90 percentiles of the samples. All Cp values except *PPIA *(P = 0.339) and *TBP *(P = 0.257) significantly differed between non-malignant and malignant samples (P of 0.0089 to < 0.0001; Wilcoxon test).

Paired malignant and non-malignant samples were always measured in the same analytical run to exclude between-run variations. Expression levels of the 10 investigated candidate reference genes (Table 2) shown in terms of Cp-values are given as box-and-whisker-plots in Figure 1. Twenty-five matched samples were included in this study. The boxes represent the median Cp-values and interquartile ranges, the whiskers indicate the 10–90 percentile ranges. Since the expressions measured in the two sample groups were not always normally distributed (D'Agostino & Pearson omnibus normality test), the non-parametric Wilcoxon signed rank test was applied for all significance calculations. Genes with higher expression levels show a lower Cp-value under the specific PCR conditions and lower expressed genes have reversely higher cycle numbers. The Cp-values of all measured genes were between 19 and 31. The genes *ACTB*, *GAPDH*, *PPIA*, *RPLPO*, *SDHA*, and *TUBB *achieved mean Cp-values between 20 and 25 cycles while the lower expressed genes *ALAS1*, *HMBS*,* HPRT1*, and *TBP *achieved Cp-values between 25 and 29 cycles. As shown in the Figure 1, all genes except *PPIA *and *TBP *showed significantly different Cp-values between the malignant and non-malignant samples. *PPIA *was the highest expressed gene with mean (± SD) Cp-values of 20.04 ± 0.97 in non-malignant and 20.04 ± 0.75 in malignant samples. The *TBP *gene was the lowest expressed gene with mean (± SD) Cp-values of 29.55 ± 0.53 in non-malignant and 29.72 ± 0.67 in malignant samples.

**Figure 2:**
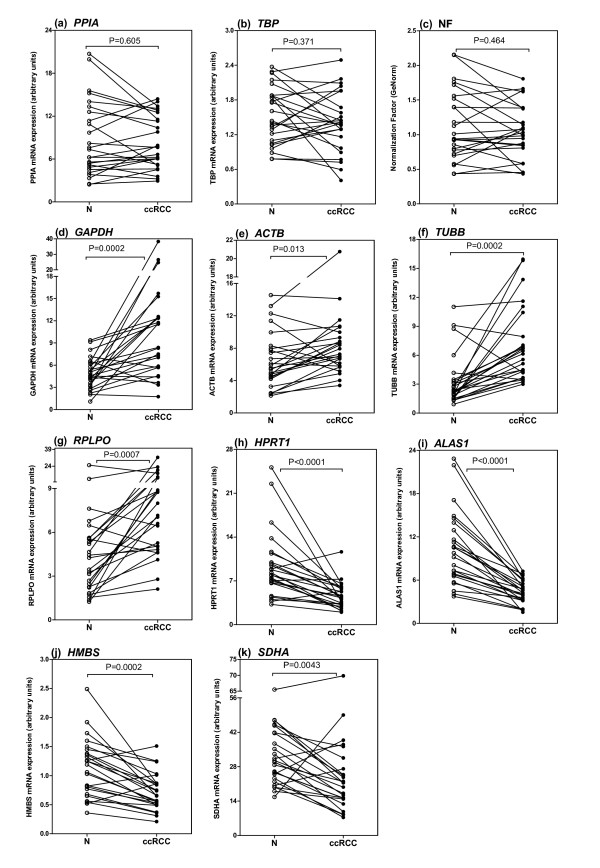
**Differential mRNA expression of candidate reference genes in matched non-malignant (N; blank circles) and malignant renal cell carcinoma (ccRCC; black circles) tissue samples shown as connected lines**. (**a-c**). Gene expression of non-regulated reference genes *PPIA *and *TBP*. The Normalization Factor was calculated from both non-regulated genes with the program geNorm (see text). (**d-g**). Gene expression of up-regulated candidate reference genes *GAPDH*, *ACTB*, *TUBB*, and *RPLPO*. (**h**-**k**). Gene expression of down-regulated candidate reference genes *HPRT1*, *ALAS1*, *HMBS*, and *SDHA*. Significances were calculated with the Wilcoxon test of paired samples.

Significance calculations between gene expression data of paired malignant and non-malignant samples were performed on the basis of concentrations instead of Cp-values, taking into account the different PCR efficiencies as indicated in Table 2. To facilitate the survey of the results, the differential expression of all 10 genes investigated is separately shown for all matched samples (Figure 2a–k). Non-regulated (Figure 2a–c), up-regulated (Figure 2d–g), and down-regulated (Figure 2h–k) genes were grouped. Significantly different gene expressions between malignant and non-malignant samples were observed for all genes with the exception of the genes *PPIA *(P = 0.605) and *TBP *(P = 0.371) (Figure 2a–c). Since the smallest mean Cp difference is 0.4 and since retrospective power calculations have yielded a high power (approx. 85%), a type II error can be ruled out with a high probability. The expressions of *ACTB *(P = 0.0132), *GAPDH *(P = 0.0002), *TUBB *(P = 0.0002), and *RPLPO *(P = 0.0007) were significantly increased in malignant compared with the matched non-malignant samples (Figure 2d–g). In contrast, the levels for *ALAS1 *(P < 0.0001), *HPRT1 *(P < 0.0001), *HMBS *(P = 0.0002), and *SDHA *(P = 0.0043) were significantly decreased in malignant tissue parts (Figure 2h–k). Thus, a statistical type I error for these eight genes is very unlikely because of the low P values. In consequence, only the two genes *PPIA *and *TBP *fulfil the essential criterion of a reference gene for gene profiling studies in renal cell carcinoma.

Except for *TUBB *and *GAPDH *the expression rates of the genes significantly correlated between the malignant and non-malignant samples (r_s _of 0.429 to 0.790; P = 0.032 to P < 0.0001). The expression of all genes in malignant and non-malignant samples did not depend on age (r_s _= -0.001 to 0.282; P = 0.996 to 0.172), sex (Mann-Whitney test; P = 0.096 to 1.00), and tumour stage (Mann-Whitney test of pT1+2 vs., pT3 or pT1 vs. pT2+3, and pT1 vs. pT3; P = 0.104 to 0.976). The effect of grading could not be estimated, since 23 of the 25 malignant samples were grade 2 and only one had grades 1 and 3.

### Expression stability testing of candidate reference genes

*PPIA *and *TBP *as the two suitable reference genes were included in the software program geNorm [4,28] to calculate a common normalization factor using the data of these two genes. The application of the normalization factor of these two genes is shown in Figure 2c; it yielded comparable data if only one of the two reference genes *PPIA *or *TBP *was used for normalization. We also included all 10 candidate reference genes in the program geNorm to calculate the average expression stability value M for all genes without excluding the genes differently expressed in the matched pairs. The ranking order from the most unstable to the most stable genes was as follows: *TUBB*, *RPLPO*, *GAPDH*, *ACTB*, *SDHA*, *PPIA*, *TBP*, *HMBS*, *ALAS1*, and *HPRT1*. The last remaining two genes *ALAS1 *and *HPRT1 *achieved a stability value of 0.426 as the most stable genes. It is remarkable that all genes had average expression stability M values less than 1.5 that is defined in the program as stability cutoff value. However, these results show that the program was not able to identify the genes with significant expression differences between malignant and non-malignant sample pairs.

Using the NormFinder program [29,30] as another free tool available on the internet to validate the expression stability of the candidate reference genes, the two genes PPIA and TBP also achieved the best stability values (Table 3). With the aid of this program, PPIA was identified as the most stable single gene with a stability value of 0.074. Thus, the stability data calculated with that program as a combined estimate of intra- and intergroup expression variations of the genes studied reflect to a certain extent the expression differences of the genes observed in the matched pairs.

**Table 3:** Candidate reference genes for normalization and their expression stability values calculated by NormFinder

Ranking order	Gene	Stability value
1	*PPIA*	0.074
2	*TBP*	0.123
3	*ACTB*	0.199
4	*HMBS*	0.301
5	*SDHA*	0.308
6	*HPRT1*	0.402
7	*GAPDH*	0.436
8	*RPLPO*	0.453
9	*ALAS1*	0.481
10	*TUBB*	0.514

A low stability value as an estimate of the combined intra- and intergroup variation of the respective gene corresponds to a high expression stability of the respective gene between the matched malignant and non-malignant samples.

### Expression levels of target gene influenced by normalization genes

**Figure 3:**
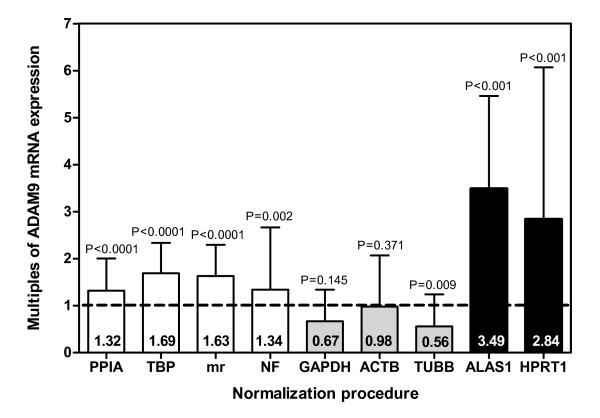
***ADAM9* mRNA expression in malignant tissue samples compared to non-malignant paired samples in depending on different normalization approaches**. Gene expression in the matched malignant tissue samples was calculated as multiple of the expression in the non-malignant sample that was set 1.0. The columns represent the median and interquartile ranges of the multiple gene expression. The four blank columns result from *ADAM9 *mRNA expression related to the two non-regulated reference genes (*PPIA, TBP*) and to the normalization factors (mr, of the two genes; NF, calculated using geNorm; see text). The three striped and two black columns illustrate the normalization of *ADAM9 *expression using either up-regulated (*GAPDH*, *ACTB*, and *TUBB*) or down-regulated (*ALAS1 *and *HPRT1*) genes.

The expression levels of the target gene *ADAM9 *is used here as an example to demonstrate the effect of different normalization genes on the relative gene expression data. We determined the *ADAM9 *mRNA expression in the same 25 RNA sample pairs as used for the reference gene search. The *ADAM9 *expressions were normalized using different strategies (Figure 3). *ADAM9 *expression values were related either to the single non-regulated reference genes *PPIA *and *TBP*, the mean ratio of both genes (mr), the normalization factor (NF) obtained for these two genes using the program geNorm, or to the three up-regulated genes *GAPDH*, *ACTB*, and *TUBB *as well as to the two down-regulated genes *ALAS1 *and *HPRT1*. To better compare the effect of these different normalization approaches, the relative *ADAM9 *mRNA expression in non-malignant samples was set 1.0 and expression rates in the matched malignant samples were calculated as multiples (Figure 3). A significant up-regulation of *ADAM9 *mRNA in malignant tissue samples was proved when the normalization approach was performed either with the two stable reference genes *PPIA *and *TBP *or with the mean ratios of both genes and the normalization factor calculated for the two genes using the geNorm program. In contrast, the normalization of *ADAM9 *expression data on the up-regulated genes resulted in a partly decreased *ADAM9 *mRNA expression, whereas the normalization procedure with the down-regulated genes resulted in an about three-fold enhancement of the up-regulation of *ADAM9 *mRNA expression in malignant tissue samples.

## Discussion

The essential result of our study evaluating 10 candidate reference genes for normalizing gene expressions of clear cell RCC was that only the two genes *PPIA *and *TBP *did not differ in their expression in malignant and in non-malignant tissue pairs. Consequently, only these two genes fulfil the criterion of expression stability between matched tissue samples and could be recommended as accurate normalizers for relative gene quantification in clear cell RCC samples. In the following, this conclusion and data will be discussed, taking into account four main aspects of the current study: (a) the particular study conditions concerning the preanalytical and analytical variables, (b) the panel of the candidate reference genes studied, (c) the validation of suitable reference genes, and (d) the limitations of the study.

### Preanalytical and analytical study conditions

Like in our previous experiments with reference genes [8,9], the preanalytical and analytical design of our study is defined by three characteristics: 1) use of paired malignant and non-malignant tissue samples from the same nephrectomized organ; 2) use of high-quality RNA samples as precondition for reliable RT-PCR measurements; 3) high analytical performance realized by measurements on the LightCycler. We consider these preconditions essential to achieve a suitable normalization for gene expression studies. Recently, Huggett et al. [17] recommended a similar approach.

The use of paired samples from the same RCC instead of unpaired samples for comparative gene expression studies has the particular advantage of minimizing the interindividual variation effect and increasing the statistical significance of the study [8,9]. The correlations between the corresponding gene expressions in the malignant and non-malignant tissue samples support the view that interindividual expression variations do occur. In consequence, differential gene expressions can be easier ascertained so that the use of paired samples results in a more exact validation regarding the suitability of reference genes than a study using unpaired samples. Therefore, the use of paired samples is the method of choice in identifying suitable reference genes for differential gene studies between malignant and non-malignant samples. In clinical research the detection of different gene expression levels between non-malignant and malignant tissues is an important objective. The results are starting points for further studies on translational stage with the final goal of developing new diagnostic markers or therapeutic approaches. However, in validating of suitable reference genes, one should above all, consider that the expression of candidate reference genes can be influenced by physiological factors like age, sex, pathological factors like the tumour characteristics stage and grade or other biological conditions [23,29,31-34]. The results of our data proved that the expression of all genes was not dependent on age, sex, and tumour stage.

Another characteristic of our study was the use of high-quality RNA samples as an important prerequisite to obtain reliable RT-PCR results [17,35] for selecting stable reference genes. For expression measurements, we used only RNA samples with an A_260_/A_280 _ratio > 1.95 and RIN values > 7.0, as an index of high purity and integrity of the RNA samples. We controlled the quality of each RNA sample with the Agilent 2100 Bioanalyzer. The principle of the bioanalyzer technology is based on microcapillary electrophoresis [27,36]. Electropherograms and gel-like images can be visually evaluated and an expert software can generate an RNA integrity number (RIN). The RIN algorithm evaluates six regions of the electropherogram in addition to the conventional ratio of 28S rRNA to 18S rRNA [27,36]. The RIN allowed a user-independent assessment of RNA integrity. Although mRNA only accounts for 2–5% of total RNA, the RIN value provides a relevant information concerning downstream applications [35]. Based on the correlation analysis of RIN values to RT-PCR data, Schroeder et al. [27] defined for their experiments a RIN value of 6 as a threshold for high and low RNA quality while the low quality RNA was considered unacceptable for downstream measurements. Collection, storage, and processing of the tissue samples are the essential preanalytical variables that affect the RNA integrity as a global indicator of all detrimental processes occurring during the time between the collection of samples after surgery and isolation of RNA [27,37]. The isolated high-quality RNA in our experiments and the proof of the non-correlation of RIN values with the expression results confirmed that possible distorting effects by non-intact RNA could be avoided. In addition, the use of RNA samples with similar degrees of integrity is shown in our experiments by the strong correlation of the paired RNA samples (r_s _= 0.635, P < 0.001). There was a mean RIN difference of only about 0.7 that minimized the variability of the RNA content in the cDNA synthesis followed by more stable quantitative RT-PCR data. In fact, Auer et al. [38] suggested that samples of comparable RNA intactness could be used for microarray analysis despite a certain degradation. Our previous experiments have shown that not only the interval time between the removal of the organ and preservation, but also the method of preservation is decisive for the integrity of RNA. Comparisons of RIN values measured in RNA isolated from paired tissue samples stored in RNAlater solution or snap-frozen in liquid nitrogen immediately after surgery showed significantly higher values using RNAlater-preservation. Therefore, we consider the use of RNAlater solution as method of choice in preservating renal tissue for obtaining intact RNA for reliable expression results. RNAlater was also recently recommended for preserving RNA integrity in routine clinical kidney biopsy material [39].

The analytical performance of the RT-PCR measurements was characterized by low variation coefficients of Cp-values ranging from 0.17 to 0.38% in between-run precision experiments. This high analytical precision, together with the use of paired samples and their measurement in the same analytical run, enabled us to carry out data analysis with high statistical probability.

### Panel of the candidate reference genes studied

As briefly outlined in the Introduction, a systematic study concerning the suitability of reference genes for normalization in RCC expression studies has been lacking so far. Schmid et al. [7] performed a Medline search about reference genes used in renal tissue in general. Although, as reported, we included in our PubMed search only publications concerning RCC (Table 1), we, like Schmidt et al. [7], found *GAPDH *and *ACTB *to be the most frequently used genes for normalization. It should be mentioned that the search strategy in PubMed using the MESH terms "renal cell carcinoma" in conjunction with the terms "RT-PCR" and "gene expression" was limited, since published studies not indexed with these terms cannot be found. The reviewer drew our attention to this point after our study was finished. For example, the study by Janssens et al. [40] using the gene mitochondrial ATP synthase 6 (*mATPsy6*) in addition to *GAPDH *and *HMBS *(named by the authors as *PBGD*) as reference genes in various cell lines and tissues was not found with this strategy and was not included in this study. However, we believe that despite this limitation, the overview in Table 1 served as good starting point for our study. The selection of eight out of the 10 candidate reference genes in our study resulted from this search to facilitate comparison, while the two additional genes *TUBB *and *SDHA *were included into the panel of candidate reference genes due to their utility shown in other studies [6,8,9]. All these candidate reference genes studied are widely used in other studies. Our study was performed to search for stable housekeeping genes in a large panel of candidate reference genes covering a broad expression range. The two genes 18S-ribosomal RNA and beta-2-microglobulin also commonly applied as normalizers in RCC samples (Table 1) were not included in our study panel because they belong to the highly expressed and regulated genes in renal tissue [6]. Moreover, the use of 18S-ribosomal RNA as reference gene would be only possible if random primed reverse transcription was carried out for cDNA synthesis.

### Validation of the suitable reference genes

To identify the suitable reference genes as normalizers in gene expression studies, several strategies including computer programs have been recommended [1,17,23,29,40-43]. There is no doubt that the absence of the differential expression of the candidate reference gene examined in the study groups in question or under conditions to be compared is the strongest proof of suitability. Therefore, we proposed to use a two-step strategy for identifying suitable reference genes according to our study design of matched malignant and non-malignant samples [8,9]. First, the expression of the candidate reference genes between the respective study groups or conditions is compared using the appropriate paired tests. It can be assumed that genes with significantly different expressions are not suited to target gene normalization, since they are affected by the study condition in question. Those genes should be excluded as normalizers. In the current study, the expression of all genes except that of *PPIA *and *TBP *varied in the matched malignant and non-malignant samples. The difference in expression was observed not only in the two genes *ACTB *and *GAPDH*, which are most frequently used as normalizers in RCC studies, but also in the gene *HPRT1*. The latter was recently recommended as single reference gene for gene expression studies in cancer research [44]. It was also identified as a suitable reference gene in RCC samples by Haller et al. [23] using the equivalence test. However, this discrepancy to our results might be due to the fact that Haller et al. [23] only studied 10 paired samples with an obviously lower statistical power compared with that in our experiments with 25 paired samples.

In the second step of our approach, we generally calculated the best-performing reference genes using the geNorm and NormFinder programs [8,9]. Whereas in prostate cancer and bladder cancer experiments 13 out of 16 and six out of nine genes, respectively, remained as suitable genes for the further calculations with geNorm and NormFinder [8,9], only the two genes *PPIA *and *TBP *could be considered for these analyses in the current study. It is worth mentioning that the geNorm program was unable to detect candidate reference genes that were characterized as unsuitable reference genes in the first step of suitability testing. In contrast, NormFinder recognized *PPIA *and *TBP *as the two best-performing reference genes when all candidate reference genes were included for the calculation (Table 3).

A target gene normalization was used as an example to illustrate the validation of a suitable reference gene selected from a panel of candidate reference genes. We used "a disintegrin and metalloproteinase domain 9" (*ADAM9*) as the target gene. Without going into details about the possible significance of *ADAM9 *in RCC tumourigenesis, we can say that the various relative quantification methods shown here can result in serious gene quantification errors if unsuitable reference genes are used (Figure 3). We recommend the use of *PPIA *and *TBP *for normalizing expression results using the mean ratio of relative quantification with the two reference genes. Although the advantage of using both reference genes for normalizing is not clearly evident in our study, other authors showed a more accurate normalization when more than one reference gene was used [4,7,33].

### Limitations of this study

Some limitations of this study should be mentioned. The first limitation could be seen in the limited number of samples used in the current study. In contrast to this limitation, it is remarkable that even with the possibly low statistical power due to the limited number of samples, clearly significant results of eight candidate reference genes were obtained. Thus, as explained in the previous section, the risk of a type II error and also of a type I error is rather unlikely as the problem with small studies does not exist in our study. Second, the present study is limited to the clear cell RCC subtype. However, that type is the most frequent malignant RCC. The expression of the two recommended reference genes *PPIA *and *TBP *in the papillary or chromophobe RCC subtypes was not studied and it would be necessary to confirm their potential use in further studies. Third, the study was partly unbalanced with regard to sex, tumour stage, and grade. The study included 21 males, but only 4 females. However, since there were no differences for all genes studied, we can assume, despite this unbalanced design, that the expression did not depend on sex. Similarly, the tumour stage obviously did not influence the expression level as shown in the Results. Twenty-three out of 25 samples had the tumour grade 2. Whereas it can be assumed that at least *PPIA *and *TBP *are suitable reference genes for grade 1, their usefulness as normalizer in grade 3 RCC samples remains to be verified. Fourth, although we examined the most comprehensive panel of potential housekeeping genes in comparison with other studies, we measured only 10 genes, excluding genes very rarely used for renal cell carcinoma due to the limited sample material available (Table 1). Thus, the question remains unanswered whether these or other genes including the mitochondrial ATP synthase 6 gene mentioned above are equivalent to or more suitable than the recommended genes *PPIA *and *TBP*.

## Conclusion

The two housekeeping genes *PPIA *and *TBP *are the sole stably expressed genes from a panel of 10 candidate reference genes studied in matched malignant and non-malignant tissue samples from the clear cell RCC. Both genes are recommended as reference genes for relative gene quantification in gene profiling studies either as single gene or preferably in combination.

## Methods

### Patients and samples

Kidney tissue samples derived from 25 adult patients with RCC (21 male, four female, mean age 62 years, range: 45 to 92 years) undergoing radical nephrectomy at the Department of Urology of the University Hospital Charité between September 2003 and January 2006. The use of the tissue material for research was approved by the Medical Ethical Committee of the Charité Hospital (Chairman: Prof. R. Uebelhack, University Hospital Charité, Berlin, Germany; protocol "Detection of metalloproteinases in patients with genitourinary cancer"; July 16, 2002). Matched malignant and non-malignant specimens from the same kidney were collected immediately after surgery in tubes with RNAlater^® ^Stabilization Reagent (Qiagen, Hilden, Germany), stored at 4°C overnight, and then put in long-time storage at -80°C until RNA isolation.

Tumour stage and classification were established according to the 2002 TNM System and the 2004 WHO Classification [12,45]. All tumours were clear cell carcinoma (ccRCC). Eleven of the 25 tumours studied were classified stage pT1, two tumours pT2, and 12 tumours pT3. The histological grading was once G1, 23 times G2, and once G3. None of the patients had metastases (M0 and pN0).

### RNA isolation and characterization

Total RNA was isolated from about 50 mg of preserved kidney tissue samples cut into small pieces and homogenized in 350 μl RNA lysis/binding buffer including 1% beta-mercaptoethanol. The RNeasy Mini Kit (Qiagen) was used for RNA isolation according to the manufacturer's instructions. An additional digestion step on the RNA binding silica gel membrane of the spin column was performed with DNase I. The RNA yield and the ratio of absorbance at 260 nm to 280 nm (A_260_/A_280 _ratio) were measured with the NanoDrop^®^ND-1000 Spectrophotometer (NanoDrop Technologies, Montchanin, DE, USA).

The integrity of isolated total RNA was assessed with the RNA 6000 Nano LabChip^® ^kit using the Agilent 2100 Bioanalyzer (Agilent Technologies, Palo Alto, CA, USA). Agilent 2100 Expert software was used to generate a so-called RNA Integrity Number (RIN) as criterion of the RNA quality for downstream experiments. The RIN values are scaled from number 1 (RNA completely degraded) to 10 (intact RNA) [27,35,36].

### First strand cDNA synthesis

One μg RNA was reversely transcribed using the Transcriptor First Strand cDNA Synthesis Kit (Roche Applied Science, Penzberg, Germany) with random hexamer priming method according to the manufacturer's recommendations. This kit was selected as result of previous comparative studies between various cDNA synthesis kits. That kit offers a fast, complete, and high-yield cDNA synthesis [46]. Briefly, RNA samples and random primers were mixed and denatured for 10 min at 65°C. Thereafter, tubes were immediately placed on ice. The first strand cDNA synthesis was started after adding transcription mixture at 25°C for 10 min (random primer annealing) following 30 min at 55°C for reverse transcriptase reaction. Finally, the enzymes were inactivated at 85°C for 5 min. Each RNA sample was controlled for genomic contamination without reverse transcriptase addition into cDNA synthesis mixture. cDNA samples were stored at -20°C and diluted 1:5 with RNase-free water for use as template in real-time PCR analysis.

### Real-time RT-PCR

Real-time PCR was performed with the LightCycler instrument (Roche) by using different measurement and detection modes. The primer and probe sequences applied for the cDNA amplification of the ten candidate reference genes and one target gene are given in Table 2. The primer/probes sequences and PCR run conditions for the detection of *ACTB*, *ALAS1*, *GAPDH*, *HMBS*, *HPRT1*, *SDH*, and *TBP *were used as previously described [9]. For *PPIA*, *RPLPO*, and *TUBB*, real-time PCR pre-designed assays (Qiagen) based on detection with SYBR Green I (SGI) fluorescence dye were used. cDNA amplification for these three genes were performed with the QuantiTect SYBR Green Master Mix (Qiagen) that contained HotStar Taq DNA polymerase, dNTPs, MgCl_2 _and a special buffer system with SGI dye. The final PCR reaction mix included 0.5 μmol/l of specific primer assay mixtures, 1 μl diluted cDNA, DNase-free water and 2 μl QuantiTect SGI PCR Master mix at a final volume of 10 μl. The cycle conditions were set for *PPIA*, *RPLPO *and *TUBB *as follows: Taq DNA polymerase activation at 95°C for 15 min with a ramping rate of 20°C/s, start of each amplification cycle with a denaturation step at 94°C for 15 s, primer annealing for *PPIA *at 55°C for 20 s and for *RPLPO *and *TUBB *at 57°C for 20 s, primer extension at 72°C for 20 s. The ramping rate was 2°C/s for all 38 or 45 PCR cycles.

The target gene *ADAM9 *cDNA amplification was performed with the ready-to-use LightCycler^® ^FastStart DNA Master^PLUS ^HybProbe (Roche). The final reaction concentrations of both primers were 0.5 μmol/l and the donor/acceptor probe concentrations were 0.2 μmol/l each. The PCR setup was: activation of FastStart Taq DNA Polymerase at 95°C for 15 min, followed by 45 cycles of denaturation at 95°C for 10 s, annealing at 62°C for 30 s and elongation at 72°C for 30 s. The temperature transition rate was 20°C/s. The final PCR volume of 20 μl included 1 μl of 1:5 diluted cDNA.

In dependence on gene-specific primer/probe sets for real-time PCRs, the amplification rate detected as fluorescence accumulation was gained by using probes after annealing phase and by using SGI after elongation step. To evaluate fluorescence data the method of Second Derivative Maximum of the LightCycler Software 3.5 (Roche) was utilized. Crossing point (Cp) values as well as quantities were applied for comparison of gene expressions in paired samples. For the quantification of each gene a pooled cDNA sample with a high expression value was stepwise diluted and amplified. The undiluted sample was set as one. The resulting standard curve was given in arbitrary units. In each PCR run a diluted secondary standard was used as calibrator and another cDNA dilution was used as run-to-run precision control. Prior to this, the control cDNA pool was adjusted in the range of sample expression level. In the same procedure the PCR efficiencies were calculated (Table 2).

Each PCR run included a no template control with water instead of cDNA. Duplicate measurements were performed and mean values were used for all further calculations. To minimize the analytical variation paired malignant and non-malignant samples were always analysed in the same PCR run.

### Data analysis

Statistical analyses were performed with GraphPad Prism for Windows, version 4.03 (GraphPad Software, San Diego, CA, USA). The distribution fitting procedure according to the D'Agostino & Pearson omnibus normality test was performed and non-parametric tests (Wilcoxon test for paired samples; Mann-Whitney test) were applied. Correlations were characterized by the Spearman's rank correlation coefficient r_s_. P < 0.05 values (two-sided) were considered as statistically significant.

To characterize the expression stability of the candidate reference genes, the programs geNorm, version 3.4 [4,28] and NormFinder [29,30] were applied. geNorm processes gene expression data as concentration values, taking into account the PCR efficiencies of investigated genes (Table 2) [4]. The most stable genes are stepwise selected from the investigated gene panel and a common Normalization Factor (NF) can be calculated for the genes selected for the normalization procedure.

The program NormFinder is a free Add-in for Microsoft Excel [29,30]. NormFinder processes data in linear scale. On the basis of a given "group identifier", the program can discriminate between different groups, e.g. malignant and non-malignant samples. The program algorithm implies the estimation of intra- and intergroup variation and combines both results in a stability value for each investigated gene. The candidate gene with the lowest stability value is the most stable gene within the groups studied. The best combination of two genes is also indicated.

## Abbreviations

A, absorbance;

Cp, crossing point;

cc, clear cell;

NF, normalization factor calculated by geNorm;

SGI, SYBR Green I;

RCC, renal cell carcinoma;

RIN, RNA integrity number;

RT-PCR, reverse transcription polymerase chain reaction

## Authors' contributions

MJ conceived the project, performed all the experimental procedures and statistical calculations, carried out drafting and writing the manuscript, and prepared the figures. AR participated in RNA isolation and RT-PCR experiments and made the PubMed search. JR, MJo, and MR contributed to the acquisition of patient's data and tissue samples after surgery, and were helpful in the interpretation of results. GK made the histopathological characterization of tissue specimens and contributed to the interpretation of data. KJ provided guidance in the study design, made special data analysis, and helped in drafting the manuscript. All authors were involved in revising critically the manuscript in progress, read and approved the final manuscript.
